# Hidden effects of dobutamine on cardiac output

**DOI:** 10.1186/s40635-026-00929-x

**Published:** 2026-07-01

**Authors:** Christopher Lai, Talal Shaikhain, Sheldon Magder

**Affiliations:** 1 Department of Critical Care Medicine, Faculty of Medicine and Health Sciences, McGill University, Montréal, Canada; 2 AP-HP, Service de Médecine Intensive-Réanimation, Hôpital de Bicêtre, DMU 4 CORREVE, Inserm UMR S_999, FHU SEPSIS, CARMAS, Université Paris-Saclay, 78 Rue du Général Leclerc, 94270 Le Kremlin-Bicêtre, France

**Keywords:** Cardiac output, Inotropes, Mean systemic pressure, Venous capacitance, Venous compliance, Shock

## Abstract

**Background:**

Dobutamine exerts its pharmacological effects by stimulating α1-, β1-, and β2-adrenergic receptors. Activation of these receptors increase both the force of cardiac contraction (inotropy) and heart rate (chronotropy). Despite its well-established cardiac effects, the influence of dobutamine on vascular tone remains poorly understood and its impact on venous return and the underlying determinants of this process has not been thoroughly investigated.

**Methods:**

In eight healthy pigs, we measured cardiac output (CO) by pulmonary thermodilution, right atrial pressure (RAP) and arterial pressure. We measured mean circulatory filling pressure (PMSF), by transiently inflating a balloon in the right atrium and thus temporarily stopping blood flow and allowing venous pressure to plateau. After obtaining baseline measurements, dobutamine was administered at 10 and 20 µg/kg/min. Two fluids bolus were given at baseline and with a dobutamine infusion at 20 µg/kg/min to produce cardiac function curves. Repeated measures ANOVA were performed for comparisons.

**Results:**

Dobutamine increased CO from 3.4 ± 0.3 to 4.3 ± 0.4 (p = 0.0145) and 4.5 ± 0.5 L/min/m^2^ (p = 0.0163) at 10 and 20 µg/kg/min, with an upward shift of the cardiac function curve, indicating improved inotropy. Stroke volume and RAP did not change, but heart rate increases linearly with the dosage of dobutamine (p = 0.0009). Mean arterial pressure did not change but systemic vascular resistance decreased (p < 0,0001). PMSF also increased with dobutamine from 8.7 ± 0.7 to 9.8 ± 0.8 and 10.4 ± 0.8 mmHg at 20 µg/kg/min (p = 0.0455), and the pressure difference for venous return (PMSF-RAP) increased from 5.6 ± 0.6 to 7.3 ± 0.6 at 10 µg/kg/min and 7.0 ± 0.5 mmHg at 20 µg/kg/min (p = 0.0292). There was no change in venous compliance or resistance to venous return, thus indicating a reduction in venous capacitance. No time-effect of dobutamine was detected over a 2-h period.

**Conclusion:**

Dobutamine increased cardiac output by increasing cardiac function by increase heart rate but also by improving venous return by increasing PMSF by decreasing venous capacitance and lowering arterial vascular resistance, underscoring the importance of its α1 and β2-adrenergic activity.

**Supplementary Information:**

The online version contains supplementary material available at 10.1186/s40635-026-00929-x.

## Introduction

This report was triggered by a recent patient we managed who had a low cardiac index and low right atrial pressure (RAP) following aorto-coronary bypass surgery that did not respond to volume. The treating physician started a dobutamine infusion despite the central venous pressure (CVP) still being low rather that giving more volume. A Dobutamine infusion was started, and cardiac index (CI) rose but unexpectedly, RAP rose rather than decreased as expected with the increase in inotropy. This result was similar to previous unreported data from an animal study at our institution which we now present to explain what we believe happened in this patient.

Dobutamine is considered a first-line inotropic agent for management of depressed cardiac function [1, 2]. Its inotropic effect occurs by binding of its ( +)-enantiomer on β1- and β2-adrenergic receptors [3]. The β1 component can raises CI by increasing inotropy and chronotropy, whereas β2 activation can cause systemic arterial vasodilation and lowers afterload. However dobutamine also acts on α1-adrenergic receptors through its (-)-enantiomer [4]. Its (-)-enantiomer binds α1-receptors to promote inotropy and vasoconstriction, and the ( +)-enantiomer acts as an α1-antagonist, leading to vasodilation [4]. The overall effect on the circulation will thus be the balance of all these activities.

It is most often considered that dobutamine increases cardiac output by an upward shift of the cardiac function curve and a consequent decrease in RAP. This improvement in cardiac function has a “permissive” effect allowing more blood to return to the heart. A dobutamine induced decrease in SVR could also act by reducing the load on the left ventricle and thus aiding ejection. All these processes should have decreased RAP so that it was surprising that RAP rose in this patient and implies that there also was a dobutamine induced increase in venous return [5]. This could happen two ways. β2 activation could decrease the resistance to venous return (RVR). However, it also is possible that activation of α1-receptors by the (-)-enantiomer decreased vascular capacitance which increased PMSF and increased venous return. A previously unpublished animal study allowed us to resolve these issues.

## Clinical case description

An otherwise healthy male except for treated hypertension, underwent a three-vessel aortocoronary bypass procedure. His pre-operative echocardiogram was normal. Surgery was uneventful and he was extubated five hours after surgery. A pulmonary artery catheter was in place as is the standard for the unit. Patient was admitted in ICU. Echocardiogram was normal immediately post-operative and patient was extubated rapidly. During the first evening his CI decreased from 2.30 L/min/m^2^ post-operatively to 1.35 L/min/m^2^, and he became oligo-anuric (Table 1). There was no evidence of bleeding. Despite receiving 2 L of fluid, his CI remained low. Dobutamine was initiated at 2.5 µg/kg/min and CI normalized as did his urine output. Surprisingly, though, despite no further volume being given, his RAP also rose with the increase in CI indicating that he somehow also had a “volume effect” [5]. Dobutamine was rapidly weaned after 24 h of infusion, and the patient was discharged from the post-operative unit on day-2. No β-blocker was administered during the stay in ICU. Hemodynamic details are given in Table 1.

**Table 1:** Clinical and biological profile of the patient

	Arrive in post operative unit from operating room	H5 post-operative	Dobutamine initiated	H1 post dobutamine
Blood pressure, mmHg	110/58	120/60	130/58	130/60
MAP, mmHg	79	80	82	83
Heart rate, /min	82	88	82	80
RAP, mmHg	8	6	7	7
PAP, mmHg	32/12	33/16	37/16	29/13
CI, L/min/m^2^	2.50	1.35	2.65	2.36
SVI, mL/m^2^	30	15	32	30
SVR, mmHg.min/L	1096	2333	1304	1460
SvO_2_, %	75.5	55.5		72.8
Hg, g/dL	10.2	10.3		9.4
Lactate, mmol/L	2.2	2.2		1.6

CI: cardiac index, MAP: mean arterial pressure, PAP: pulmonary artery pressure, RAP: right atrial pressure, SvO_2_: mixed venous saturation in oxygen, SVR: systemic vascular resistance

## Materials and methods

### General methods

All procedures were conducted in accordance with the guidelines of the animal care committee at the Royal Victoria Hospital, Montreal. Domestic female pigs, weighing 27.1 ± 8.5 kg, were sedated using ketamine (30 mg/kg), atropine (1.0 mg/kg), and xylazine (2 mg/kg). After twenty minutes, anesthesia was induced with 10 to 15 mg/kg of sodium thiopental and maintained via a continuous intravenous infusion at 5 mg/kg/h. The animals were positioned supine in a V-shaped support, intubated with a cuffed endotracheal tube, and mechanically ventilated with a tidal volume of 12 mg/kg, respiratory rate of 12 to 15 breaths/min, and positive end-expiratory pressure of 5 cmH_2_O.

A midline neck incision was performed to isolate and cannulate the left common carotid artery with a catheter for arterial pressure monitoring. The right internal jugular vein was similarly isolated, and a Swan-Ganz flow-directed catheter advanced to the pulmonary artery. Additionally, a 12F balloon-tipped Prewitt aortic occlusion catheter (capacity 50 cc; Pramel Inc, Longueuil, Quebec, Canada) was inserted into the right atrium via an external jugular vein. The right femoral vein was cannulated with a polyethylene tube (Cole Palmer, Anjou, Quebec) for drug administration. Blood gas levels were regularly monitored, ensuring that PCO_2_ remained between 35 and 40 mmHg through ventilator adjustments and PO_2_ above 90 mmHg by providing supplemental oxygen. Cardiac output (CO) was assessed using the pulmonary thermodilution method (Abbott 3300, North Chicago, IL) by injecting 3 mL of 5% dextrose in water at room temperature into the right atrial port of the Swan-Ganz catheter.

### Measurements of PMSF

Mean systemic filling pressure (PMSF) was measured by rapidly inflating the balloon in the right atrium with 40 cc of air for 15 to 20 s. This temporarily stopped venous return and venous pressure reached a plateau [6, 7]. In the absence of flow, the pressure recorded in the central vein corresponds to the upstream pressure in the compliant region of the venous system. The procedure could be repeated multiple times without affecting the animal's hemodynamic parameters or overall condition. Reproducibility was demonstrated, with repeated measurements under identical conditions differing by less than 0.5 mmHg [8].

### Vascular compliance and cardiac function curve

Compliance is the change in volume for a change in pressure and is defined as the inverse slope of the pressure–volume relationship. To assess vascular compliance, PMSF and CO were first measured. A rapid infusion of 5 mL/kg of colloids (Dextran), was then given followed by repeat measurements of PMSF and CO. This process was repeated with another 5 mL/kg of colloids for a total of 10 mL/kg. Compliance was calculated based on the change in volumes (5 and 10 mL/kg), and the associated increases in PMSF. Because veins are much more compliant than arteries, these measurements primarily reflect venous compliance. The three data points were used to plot the pressure–volume relationship between PMSF and blood volume and an evaluation of changes in capacitance by the position of the curve and changes in compliance from the slope of the curve. The relationship between CO and RAP before and after the fluid boluses (at 5 and 10 mL/kg) were used to generate cardiac function curves. After the measurements, 10 mL/kg of blood was withdrawn to avoid fluid accumulation.

### Hemodynamic calculations

The systemic vascular resistance (SVR) was calculated as SVR = (MAP—PMSF)/CO, where MAP is the mean arterial pressure. Pulmonary vascular resistance (PVR) was calculated from PVR = (mean PAP—PAOP)/CO, where PAP is the pulmonary artery pressure and PAOP is the pulmonary artery occlusion pressure. Resistance to venous return (RVR) was calculated from RVR = (PMSF—RAP)/CO, as CO equates venous return under steady state conditions. All these are in the units of mmHg.min/L.

### Protocol

**Fig. 1:**
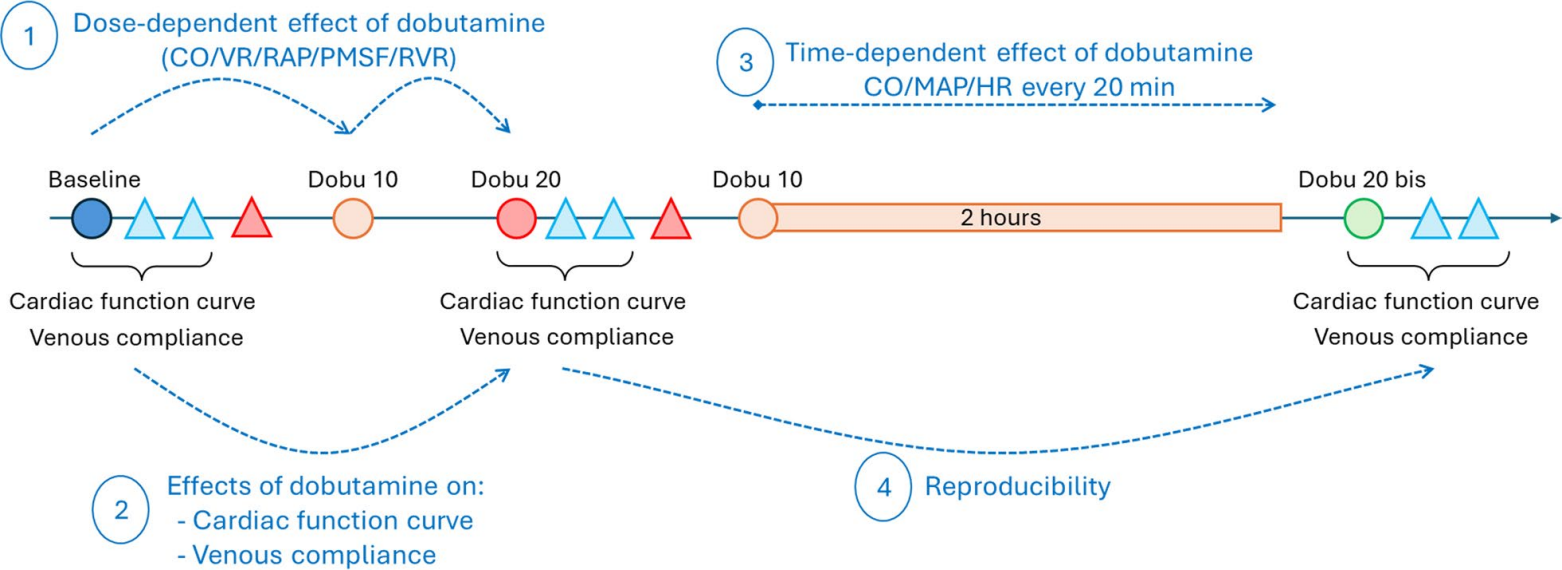
Description of the protocol and different analysis performed in animals. Blue triangle: fluid bolus of 5mL/kg of Dextran; red triangle: blood withdrawal of 10mL/kg. CO: cardiac output; Dobu: dobutamine; HR: heart rate; MAP: mean arterial pressure: PMSF: mean systemic filling pressure; RAP: right atrial pressure; RVR: resistance venous return; VR: venous return

After a 30-min period of stabilization, initial measurements of the cardiac function curve and venous compliance were performed by rapidly administering the two times 5 ml/kg bolus (Fig. 1). Then 10ml/kg of blood were withdrawn. Dobutamine was started at 10 µg/kg/min and then increased to 20µg/kg/min. New measurements of the cardiac function curve and venous compliance were done (Fig. 1). When hemodynamics stabilized, dobutamine was infused at 10 µg/kg/min for 2 h, with evaluation of hemodynamics every 20 min. After 2 h, we increased dobutamine to 20 µg/kg/min again. Hemodynamic measurements with the cardiac function curve and venous compliance were again repeated. To avoid effects of fluids and an increase in cardiac preload, 10ml/kg of blood were withdrawn after each step necessitating the fluid administration (Fig. 1).

The study consisted of four predefined stages (Fig. 1): (1) evaluation of dose-dependent effects of dobutamine (baseline, 10 µg/kg/min, 20 µg/kg/min); (2) assessment of cardiac function and venous compliance with and without dobutamine; (3) evaluation of potential time-dependent effects during a 2-h infusion at 10 µg/kg/min; and (4) assessment of reproducibility by comparing the two measurements performed at 20 µg/kg/min.

Hemodynamic assessment was performed 20 min after each dobutamine dose change to confirm stabilization and adequate drug effect or washout.

We evaluated the hemodynamic effects of dobutamine doses of 10 and 20 µg/kg/min as it represents dose range recommended in recent guidelines [9] and are expected to produce sufficient pharmacological effects while also limiting repeated measurements.

### Statistical analysis

The normality of the data was assessed using the Shapiro–Wilk test. All variables met normality distribution. All values are presented as mean ± standard deviation for descriptive variables and mean ± standard of error of the mean for repeated-measures and figures. A one-way ANOVA for repeated measurements was used to evaluate significance differences among conditions or over time, and where significance was found, post-hoc analysis was performed with a Wilcoxon signed-rank test with a Bonferroni correction. Linear trend analyses were performed using the linear-trend component of the repeated-measures ANOVA. For comparison of cardiac function curves, we performed an ANCOVA with cardiac output as the dependent variable, dobutamine administration as a factor and fluid administration as a covariate. A p-value < 0.05 was considered significant. The statistical analysis was performed using MedCalc 19.2.1 software (MedCalc Software Ltd., Ostend, Belgium).

## Results

### Animal study

#### Cardiac output, stroke volume et heart rate

Dobutamine infusion from 10 to 20 ug/kg/min produced a dose-dependent increase in heart rate. At 10 µg/kg/min, heart rate rose significant compared with baseline and increased further with 20 µg/kg/min, but stroke volume did not change significantly. As a result, there was a linear trend in CO (Table 2). MAP remained constant with no differences across doses. SVR decreased significantly in a dose-dependent relationship (Table 2).

**Table 2:** Dose-dependent hemodynamic effects of dobutamine

	Baseline	Dobutamine 10 µg/kg/min	Dobutamine 20 µg/kg/min	p
Cardiac output, L/min	3.4 (2.8–3.9)	4.3 (3.4–5.3)*	4.5 (3.3–5.7)*	0.001
Stroke volume, mL	24 (19–29)	22 (15–29)	21 (13–30)	0.242
Heart rate, /min	145 (123–168)	208 (169–248)*	226 (189–264)*^$^	< 0.001
Mean arterial pressure, mmHg	101 (94–109)	106 (96–116)	94 (82–106)	0.108
Mean pulmonary artery pressure, mmHg	16 (12–19)	17 (13–21)	18 (14–21)	0.177
Pulmonary artery occlusion pressure, mmHg	3 (0–5)	2 (0–3)	2 (0–3)	0.124
Right atrial pressure, mmHg	3.4 (1.5–5.3)	2.5 (0.7–4.3)	3.4 (1.9–4.8)	0.352
Mean circulatory filling pressure, mmHg	8.3 (7.0–9.6)	10.0 (8.1–11.8)	10.4 (8.6–12.2)*	0.002
Venous pressure difference, mmHg	5.6 (4.1–7.0)	7.4 (5.8–9.1)*	7.0 (5.9–8.1)	0.003
Systemic vascular Resistance, mmHg.min/L	2504 (2018–2992)	2014 (1604–2424)*	1673 (1366–1980)*	< 0.001
Venous return resistance mmHg.min/L	138 (98–179)	142 (112–172)	129 (106–152)	0.527
Pulmonary vascular Resistance, mmHg.min/L	318 (234–401)	296 (199–393)	298 (213–383)	0.521

^*^p < 0.05 vs baseline

^$^p < 0.05 vs dobutamine 10 µg/kg/min

Values are given as mean (95% confidence intervals)

### Venous return and PMSF

**Fig. 2:**
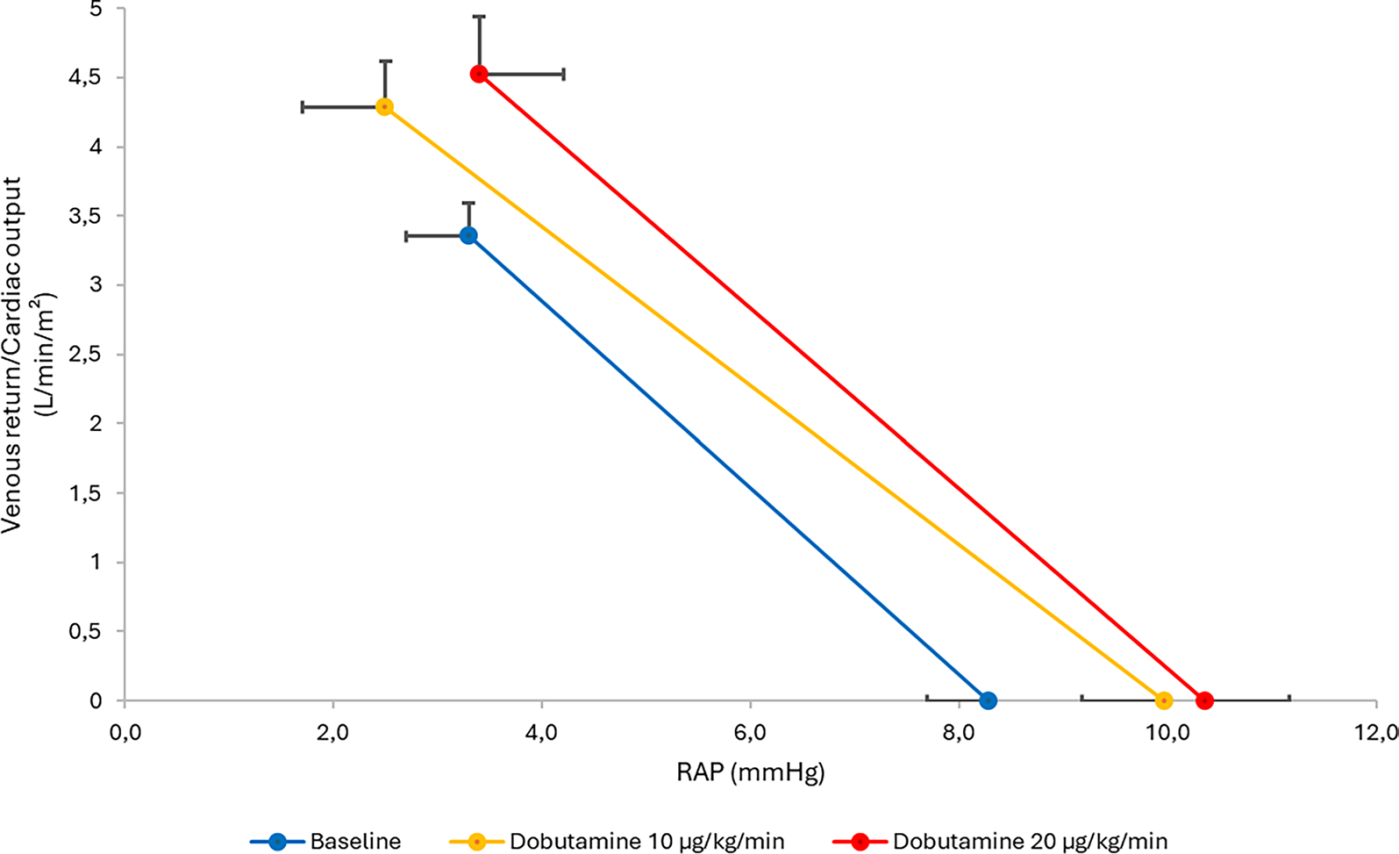
Venous return curves at different dose of Dobutamine. Values are represented as mean and standard error of the mean

Dobutamine shifted the venous return curve to the right, with no change in slope (Fig. 2). RAP did not change with dobutamine at the different dosages. Both PMSF and the pressure difference for venous return increased with dobutamine in a statistically significant linear trend (Table 2, Fig. 2). RVR did not change significantly at any of the dobutamine doses (Table 2, Fig. 2).

### Venous compliance

**Fig. 3:**
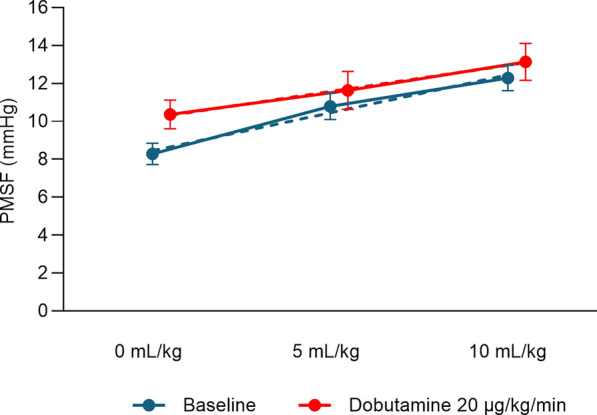
Changes in mean systemic filling pressure with changes in volume without and with Dobutamine at 20 µg/kg/min. Dotted lines are the linear regression of the changes in PMSF induced by fluid bolus. The slope of the regression line represents venous compliance. Values are represented as mean and standard error of the mean

At baseline without dobutamine, PMSF increased linearly from 8.3 ± 0.6 mmHg to 10.8 ± 0.7 mmHg (p < 0.001) and 12.3 ± 0.7 mmHg (p < 0.001) with 5 mL/kg and 10 mL/kg boluses of fluid (r^2^ = 0.980, p < 0.001). The pattern was the same with dobutamine 20 µg/kg/min indicating no change in compliance. With dobutamine 20 µg/kg/min, PMSF increased linearly from 10.4 ± 0.8 mmHg to 11.6 ± 1.0 mmHg (p < 0.001) and 13.1 ± 1.0 mmHg (p < 0.001) with 5mL/kg and 10mL/kg boluses of fluid (r^2^ = 0.996, p < 0.001) (Fig. 3). Dobutamine at 20 µg/kg/min also induced an upward shift of the venous pressure–volume curve indicating a decrease in capacitance.

### Cardiac function curve

**Fig. 4:**
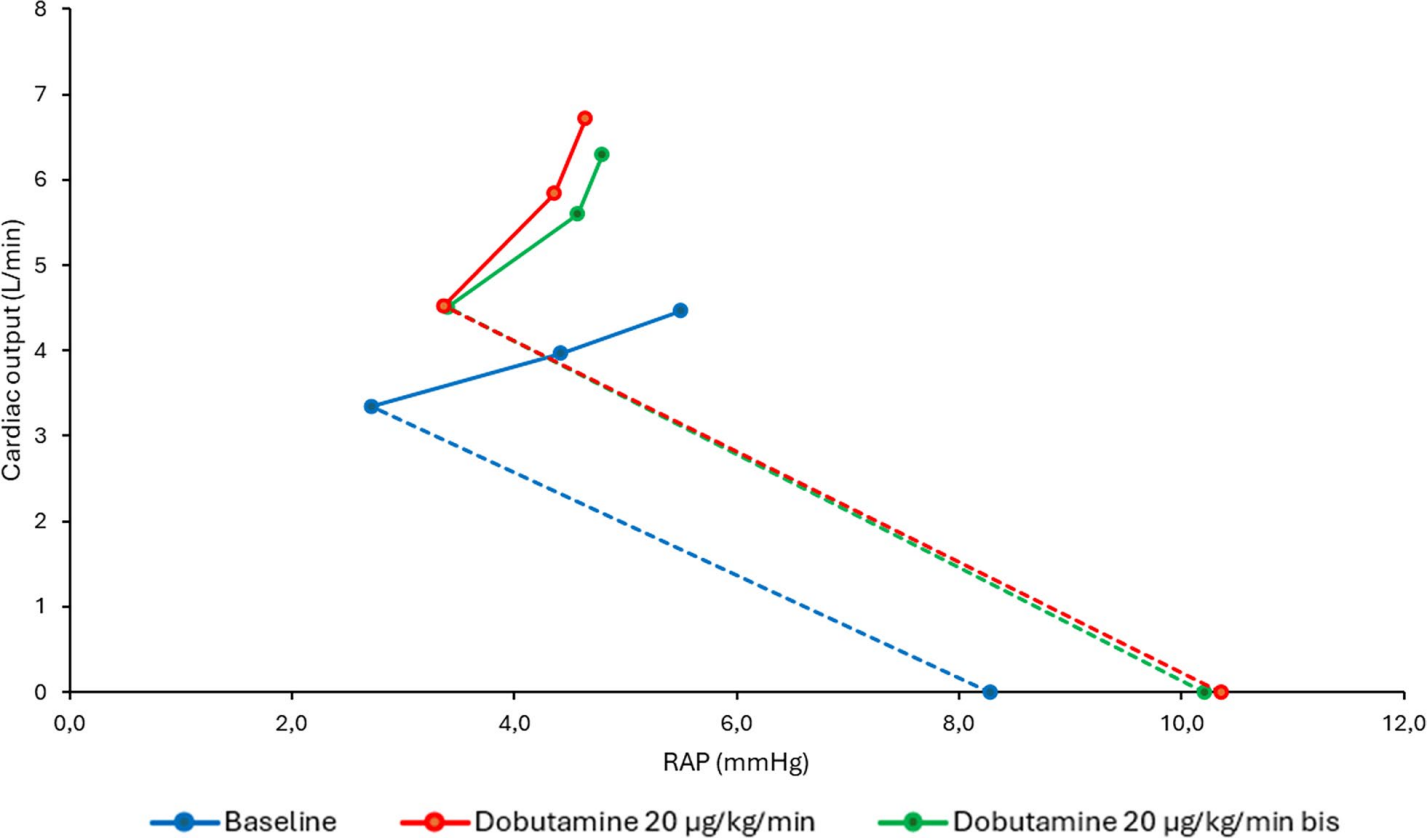
Relationship between cardiac index and right atrial pressure (RAP) without and with dobutamine at 20 µg/kg/min. Dotted lines represent venous return curves and solid lines represent cardiac function curves. The x-intercept represents mean systemic filling pressure. Values are represented as mean

The 5mL/kg and 10mL/kg boluses increased CO at baseline from 3.4 ± 0.3 L/min to 4.0 ± 0.3 L/min (p = 0.003) and 4.5 ± 0.3 L/min (p < 0.001) and CO increased similarly with dobutamine at 20 µg/kg/min from 4.5 ± 0.5 L/min/m^2^ to 5.9 ± 0.7 L/min/m^2^ (p = 0.008) and 6.7 ± 0.6 L/min/m^2^ (p < 0.001)(Fig. 4, Figure S1). Dobutamine 20 µg/kg/min also produced an upward shift of the cardiac function curve and an increase in the slope (ANCOVA; p < 0.001), indicating an increase in cardiac function (Fig. 4).

### Effects on PAP and PVR

Mean PAP (linear trend p = 0.0992) and PAOP (linear trend p = 0.1192) were unchanged with dobutamine (Table 2). There was a linear dose dependent increase in transpulmonary gradient (mean PAP-PAOP) from 12.9 ± 1.3 mmHg at baseline to 15.1 ± 1.8 mmHg and 16.0 ± 1.5 mmHg at 10 and 20 µg/kg/min of dobutamine respectively (ANOVA, p = 0.036). PVR remained unchanged (Table 2).

### Time-effect of Dobutamine

Data from the six repeated measurements were available in six animals. Analysis of time-effects of prolonged infusion of dobutamine (20 to 120min) at 10 µg/kg/min was available in six animals and showed no-significant increase in SV (ANOVA p = 0.058) or CO (ANOVA p = 0.077), heart rate, CVP, or MAP over the 2-h (Supplemental materials, Figure S2-S4).

## Discussion

Dobutamine increased venous return in healthy pigs. It occurred by a dose dependent increased PMSF without a change in RVR or venous compliance. Cardiac function also improved in the animals by a dose-dependent increase in heart rate without a change in stroke volume. There were no significant changes in pulmonary or systemic circulatory parameters except for a decrease in SVR. The observations in the pigs were similar to the clinical case except that in the patient SV improved and heart rate did not change.

Dobutamine is a racemic mixture of two enantiomers, each contributing differently to its hemodynamic effects [3]. The (-)-enantiomer primarily stimulates α1-adrenoceptor, causing vasoconstriction in capacitance vessels such as the splanchnic veins and enhances venous return. The ( +)-enantiomer stimulates both β1- and β2-adrenoceptors, increasing inotropy and chronotropy, while β2 activation can also cause systemic vasodilation. The vasoconstrictive effect of the (-)-enantiomer on capacitance vessels likely contributed to the observed increase in venous return, while the β1- and β2-mediated effects of the ( +)-enantiomer explain the enhanced cardiac function without significant changes in venous compliance.

Our study is unique in that it evaluated both the venous return function and the cardiac function curve. According to Guyton’s framework, CO and venous return must be equal at steady state [10], but they are determined by two independent physiological functions whose intersection defines the actual flow. The cardiac function curve reflects the CO against RAP relationship, whereas the venous return function is defined by PMSF, RAP, and RVR. Distinguishing CO from the cardiac function curve, and VR from the venous return function, is essential for interpreting dobutamine’s hemodynamic effects.

### Effects on venous return and its determinants

To our knowledge, this is the only study that has evaluated dobutamine’s effects on venous return function. The venous return is determined by the pressure difference between PMSF and RAP, and RVR [11, 12]. Dobutamine increased venous return primarily through an increase in PMSF, without changes in RAP or RVR. Because PMSF reflects stressed volume and venous capacitance [13], this pattern indicates a reduction in venous capacitance rather than a change in venous resistance. Stop-flow measurements allowed true determination of PMSF under static conditions, avoiding the mathematical bias in calculating venous return from CO alone. Thus, the observed rightward shift of the venous return curve represents a genuine vascular effect of dobutamine.

The primary change in venous return was an increase in PMSF which is physiologically consistent with an α₁-mediated recruitment of unstressed volume from the splanchnic reservoir into stressed volume. Venous blood comprises approximately 70% of the total blood volume, with the majority residing as unstressed volume in the splanchnic reservoir. Unstressed volume fills the veins without generating pressure, while stressed volume stretches the venous walls and provides the potential energy necessary for blood flow. Because most venous blood is located in the splanchnic reservoir as unstressed volume, α1-receptor stimulation plays a crucial role in mobilising this blood into stressed volume, thereby increasing venous return [14]. In a study of 26 dogs, Fuchs et al. showed that dobutamine infusion caused spleen contraction and reduced spleen weight by up to one third at 16 µg/kg/min [15]. This effect was reversed with α-blockade, indicating that dobutamine increases venous return and cardiac output via α-adrenergic-mediated reduction in vascular capacitance [15]. This aligns with our findings: dobutamine was associated with decreased venous capacitance and increased PMSF. Observations of unchanged or even increased RAP with improved CI have been made in other studies [16–18], but only a true assessment using stop-flow measurements could confirm this effect on the venous side and nicely explains the rise of RAP in our patient and animals, thereby improving venous return pressure gradient and venous return.

RVR remained unchanged in the animals. This may reflect the balance between α- and β-receptor stimulation with dobutamine. However, this result must be interpreted with caution. RVR is calculated using PMSF, RAP, and CO measurements, which may introduce noise and large variance in the calculation. We cannot exclude the possibility of a small change in RVR that was not detectable due to the large variance and our limited sample size. Given our small sample size and potential interspecies differences in receptor distribution and sensitivity [19], caution is warranted when extrapolating these results to broader populations or other species.

Dobutamine did not alter venous compliance. In a previous study, blockade of α-adrenergic receptors at low blood pressure increased unstressed volume, revealing that capacitance had decreased. In contrast, venous compliance did not change [20]. Dobutamine, by stimulating α-receptors, may therefore decrease capacitance without changing compliance.

### Effects on cardiac output and its determinants

Since venous return and cardiac output are equal at equilibrium [10, 12, 13], increasing venous return will likewise increase cardiac output. In our study, dobutamine infusion was associated with a marked increase in CO, primarily driven by a dose-dependent increase in heart rate. Despite this chronotropic effect, stroke volume did not significantly change, indicating that the enhancement in cardiac performance was due to frequency rather than volume per beat at the doses studied in contrast to the patient in which CO rose by an increase in stroke volume. Dobutamine was administered at 10 and 20 µg/kg/min in our study and only at 2.5 ug/kg/min in the patient so that it is possible that dobutamine’s hemodynamic effects vary with dose as previously described [20–22]. In stress echocardiography, too, cardiac output increases by increased stroke volume at lower does whereas at higher doses—typically above 10 µg/kg/min—it increase by increases in heart rate [23]. More data need to be collected from patients based on the dose of dobutamine in order to confirm this potential biphasic effect.

But chronotropy alone cannot explain the increase in PMSF. PMSF is determined by vascular tone and venous capacitance, not by heart rate or contractility. Moreover, an isolated increase in cardiac function should have produced a decrease in RAP. This decrease in RAP would continue until RAP reached zero pressure in which case there would be no further increase in CO or venous return without a change in the venous return function. The fact that RAP remained unchanged despite a substantial rise in CO is therefore physiologically meaningful: it indicates that venous return function increased in parallel with cardiac function, and that this increase could not have been achieved by cardiac stimulation alone. This distinction reinforces that dobutamine’s effects on venous return function and cardiac function are complementary but mechanistically distinct.

Taken together, dobutamine shifted both the venous return curve (rightward, via increased PMSF) and the cardiac function curve (upward). Because flow is determined by the intersection of these two curves, the combined effect explains the observed increase in CO despite stable RAP and unchanged venous resistance.

### Clinical implications

Dobutamine distinct receptor activities interact to produce a complex haemodynamic profile that may depend on the patient’s clinical status and baseline sympathetic activity. As our experimental data show, dobutamine can increase venous return by reducing venous capacitance, but this effect may be attenuated or reversed when α-receptor responsiveness is impaired [24] as well as the subjects volume status. In sepsis, alpha-receptor responsiveness is reduced (down-regulation) [25], requiring higher doses of dobutamine to achieve improved inotropy, which may shift the response toward beta-mediated vasodilation. The latter having no effect on venous capacitance but could decrease RVR [26], and increase venous return, highlighting the importance of monitoring haemodynamic when using dobutamine in different clinical contexts [27]. The specific distribution of adrenergic receptor subtypes also varies between humans and animal models, and across vascular beds, further influencing the systemic response to dobutamine [19].

Importantly, these changes may not be apparent if only heart rate and arterial pressure are monitored. In our study, MAP remained stable and heart rate increased without a change in stroke volume, meaning that the rise in CO would have been missed without direct measurement. The observed decrease in systemic vascular resistance was associated with increased blood flow and thus could have been due to a primary β2-mediated vasodilation or a baroreceptor response keeping arterial pressure constant, underscoring the limitations of interpreting SVR in isolation.

The dose-dependent chronotropic response also raises a practical question. Once heart rate increases substantially, can clinicians still expect an improvement in stroke volume? At higher doses, the contribution of stroke volume becomes limited, and increases in cardiac output may rely almost entirely on tachycardia. This reinforces the need to titrate dobutamine based on measured flow rather than assumed physiological responses [27].

### Limitations

Our study has some limitations. First, we did not evaluate hemodynamic effects of dobutamine doses less than 10 µg/kg/min, which may elicit different physiological responses. Second, our study population was healthy pigs. Therefore, extrapolation to humans or disease states should be approached with caution. Hemoglobin was not systematically measured, and although anemia cannot be excluded, the reproducibility of hemodynamic responses at both 20 µg/kg/min stages suggests that any effect of anemia was minimal. We acknowledge that the small sample size and inherent biological variability limit statistical power, meaning that clinically relevant changes in MAP or RAP may not have reached statistical significance. Nevertheless, it does not affect our central finding, as PMSF showed consistent directional changes across animals, allowing clear identification of a dobutamine-induced shift in the venous return function. Finally, both the animals and the clinical case had normal systolic function at baseline. While this limits extrapolation to patients with impaired ventricular function, it is precisely this preserved contractility that allowed us to isolate and reveal the venous effects of dobutamine. An isolated increase in cardiac function would be expected to lower RAP. The fact that RAP did not decrease despite a marked rise in CO made the increase in PMSF and the shift in the venous return function clearly identifiable. In patients with systolic dysfunction, the cardiac function curve is flatter, and changes in venous return function may be more difficult to distinguish from changes in cardiac function.

## Conclusion

In conclusion, dobutamine increased forward flow not only by cardiac stimulation but also by optimising venous return dynamics, with a reduction of venous capacitance leading to an increase in PMSF without changes in RVR. Our results also underline the possible various hemodynamic effects of dobutamine, arguing for a monitoring of hemodynamic when introducing or titrating such medication [27].

## Supplementary Information

Supplementary material 1.

## Data Availability

The data set used and analysed for the study is available from the corresponding author on reasonable request.

## Abbreviations

CI: Cardiac index
CO: Cardiac output
CVP: Central venous pressure
MAP: Mean arterial pressure
PAOP: Pulmonary artery occlusion pressure
PAP: Pulmonary artery pressure
PMSF: Mean systemic filling pressure
RAP: Right atrial pressure
RVR: Resistance to venous return
SvO_2_: Mixed venous saturation in oxygen
SVR: Systemic vascular resistance
