# Supplementary material for: Hidden effects of dobutamine on cardiac output
Source: Intensive Care Med Exp. 2026 Jul 1;14:82. doi: 10.1186/s40635-026-00929-x (PMC13323774; doi:10.1186/s40635-026-00929-x)
Supplement: Supplementary file 1 — Supplementary material 1. [file 40635_2026_929_MOESM1_ESM.docx]

**SUPPLEMENTARY MATERIALS**

**Hidden effects of Dobutamine on cardiac output: an animal study.**

Christopher LAI, Talal SHAIKHAIN, Sheldon MAGDER.

Table des matières

[Figure S1. Changes in cardiac output with changes in volume without and with Dobutamine at 20 µg/kg/min. 2](#_Toc221016730)

[Figure S2. Effects of dobutamine infusion at 10µg/kg/min on cardiac output over 120 minutes. 2](#_Toc221016731)

[Figure S3. Effects of dobutamine infusion at 10µg/kg/min on central venous pressure over 120 minutes. 3](#_Toc221016732)

[Figure S4. Effects of dobutamine infusion at 10µg/kg/min on heart rate over 120 minutes. 3](#_Toc221016733)

# Figure S1. Changes in cardiac output with changes in volume without and with Dobutamine infusion at 20 µg/kg/min.

F1: first fluid bolus; F2: second fluid bolus.

Values are represented as mean and standard deviation.

# Figure S2. Effects of dobutamine infusion at 10µg/kg/min on cardiac output over 120 minutes.

# Figure S3. Effects of dobutamine infusion at 10µg/kg/min on central venous pressure over 120 minutes.

# Figure S4. Effects of dobutamine infusion at 10µg/kg/min on heart rate over 120 minutes.
